# Portopulmonary hypertension caused by portal hypertension that is complicated by central diabetes insipidus

**DOI:** 10.1002/ccr3.2675

**Published:** 2020-02-11

**Authors:** Tingting Zhao, Qian Xing, Xiaojuan Yang, Xiaofeng Yao, Ying Ba, Haicheng Zhou, Dan Liu

**Affiliations:** ^1^ Department of Nephrology The First Affiliated Hospital of Dalian Medical University Dalian China; ^2^ Department of Endocrinology The First Affiliated Hospital of Dalian Medical University Dalian China; ^3^ Department of Ophthalmology Xi'an Fourth Hospital Xi'an China; ^4^ Department of Preventive Medicine Dalian Medical University Dalian China

**Keywords:** desmopressin, idiopathic central diabetes insipidus, idiopathic portal hypertension, portopulmonary hypertension, vasopressin

## Abstract

We discuss the pathophysiology, diagnosis, differential diagnosis, and therapy of a case with central diabetes insipidus, idiopathic portal hypertension, and portopulmonary hypertension. This report reviews how vasopressin affects those diseases.

## Graphical abstract

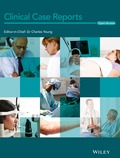

## 1 INTRODUCTION

CDI presents with polydipsia, polyuria, and dilute urine which are caused by a lack of arginine vasopressin (AVP). IPH is a hepatic disorder of unclear etiology, that is characterized by esophageal varices, splenomegaly, preserved liver function, and increased portal venous pressure. These two diseases are related via vasopressin.

## 2 CASE REPORT

In December 2017, a male patient aged 31 years visited our department with severe thirst, persistent polyuria and polydipsia. The patient developed anxiety and overwork a month prior. His fluid intake was up to 10 L/d with a commensurate urine output of >10 L/d. He was diagnosed with tuberculosis 11 years prior, and he recovered after antituberculosis therapy for 1 year. Seven years prior, he was admitted to another hospital with hematemesis and melena and was diagnosed with upper gastrointestinal hemorrhage, esophageal varices, and splenomegaly. CT portal venography and inferior vena cava venography showed portal vein and splenic vein thickening. He underwent limited side‐to‐side portal‐caval shunt placement, splenectomy, and hepatic wedge resection. Three years prior, he developed nausea, vomiting, and dyspnea, and eventually collapsed abruptly while climbing stairs. At that time, his cardiovascular examination revealed severe pulmonary artery hypertension (PAH).

No abnormality was detected from physical examination. Urine analysis revealed a specific gravity of 1.000 (normal, 1.003‐1.03), a urine osmolality of 80 mOsm/kg·H_2_O, a serum osmolality of 310 mOsm/kg·H_2_O, and no obvious abnormality in the pituitary hormone levels and perimetry. MRI showed an elliptic pituitary. T1‐weighted imaging (T1WI) revealed a low‐density lesion with a diameter of 0.5 cm in the left wing of the pituitary. Hence, pituitary microadenoma was suspected following MRI. Chest X‐ray presented plaques in the right lower lobe of the lung that were suspected to be old lesions. Ultrasonic cardiography displayed severe pulmonary hypertension with an estimated systolic pulmonary arterial pressure (sPAP) of 85 mm Hg.

A water restriction test and a desmopressin (DDAVP) trial were performed as the diagnostic “gold standard,” although pitfalls existed. After water deprivation overnight (8 hours), the patient presented a basal urine osmolality of 170 mOsm/kg·H_2_O and a serum osmolality of 325 mOsm/kg·H_2_O, reaching a plateau of urinary osmolality. Thereafter, 5 U pitressin was administered subcutaneously. The urine osmolality subsequently rose to 590 mOsm/kg after 2 hours (representing an increase in the urinary osmolality of 50% relative to the baseline level). The test was terminated at this point. Therefore, the diagnosis of complete CDI was confirmed. No routine laboratory tests or MRI images were available to indicate inflammatory diseases, tumors, or autoimmune diseases, which would indicate the differential diagnosis of CDI. CDI and pulmonary hypertension might occur in patients with pulmonary Langerhans cell histiocytosis.^1^ Nevertheless, no evidence has supported the diagnosis of Langerhans cell histiocytosis. The patient had a history of tuberculosis, but he recovered 10 years prior, and the chest X‐ray and T‐SPOT results revealed no active tuberculosis.

Regarding the differential diagnosis of portal hypertension, the patient had neither risk factors for chronic liver disease nor evidence of hypohepatia. Additionally, neither inferior vena cava angiography nor hepatovenography revealed evidence of stenosis. Simple liver biopsy revealed slightly swelling hepatocytes and a thickened portal vein wall, with no necrosis in liver lobules or cirrhosis.

For the differential diagnosis of PAH, pulmonary artery CTA revealed no pulmonary embolism. The patient developed PAH after the diagnosis of IPH, and the hyperdynamic circulatory state and high cardiac output were suggested to result in increased shear stress on the pulmonary arterial wall, leading to pulmonary vasoconstriction and vascular bed remodeling.^2^ Additionally, the patient underwent limited side‐to‐side portal‐caval shunt placement, shunting the pressure into the inferior vena cava, accelerating the progression of PAH, and increasing the cardiopulmonary pressure.

The patient was administered oral DDAVP acetate at a dose of 0.05 mg four times daily. After receiving this therapeutic regimen, his 24‐hours urine output was within the range of 6‐7 L. The patient was in a stable condition after receiving the therapy for 8 months. Meanwhile, his 24‐hours urine output was within the range of 4‐5 L when he received DDAVP at a dose of 0.2‐0.25 mg per day. However, polyuria and polydipsia returned after the discontinuation of DDAVP.

## 3 DISCUSSION

Vasopressin, the pro‐drug of lysine vasopressin, increases mesenteric vascular resistance and the mean arterial pressure but decreases mesenteric blood flow dose dependently. Therefore, it is used to control bleeding esophageal varices in portal hypertensive patients.^3^ V1 receptors can be used to mediate the contraction of systemic and splanchnic arteriolar smooth muscle, thus decreasing the portal and hepato‐splanchnic flow. DDAVP is a synthetic analog of AVP that can serve as a specific agonist of AVP V2 receptors, and it has a lower effect on blood pressure than AVP. DDAVP has been used to treat CDI. Pulmonary hypertension has been reported to aggravate the prognosis for diabetes insipidus.^4^ However, Johns RA suggested that DDAVP is a potent vasorelaxant of pulmonary arteries^5^ and DDAVP‐induced vasodilation might be related to the activation of endothelial NO synthase via Ser1177 phosphorylation.^6^ DDAVP was speculated to not worsen PAH of the patient, a finding that was also confirmed by long‐term follow‐up. Studies have shown that portal hypertension in cirrhosis can cause peripheral arterial vasodilation and AVP secretion, surpassing the inhibition of AVP release from the osmotic pathway mediated by baroreceptors and the nonosmotic stimuli.^7^ However, our patient showed a deficiency in AVP with CDI. The pathogenesis of the patient remains unknown.

## 4 CONCLUSIONS

We report the first case of CDI, IPH, and POPH with a 6‐month follow‐up and illustrate the marked effect of vasopressin on CDI, IPH, and POPH. Although rare, this case extends the views on the diagnosis and should be considered in cases of central diabetes insipidus of unclear etiology. Further investigation is warranted to understand the role of AVP in the pathogenesis of this patient. Our report should encourage studies of similar patients to identify the potential pathogenesis and better treatments.

## CONFLICT OF INTEREST

None declared.

## AUTHOR CONTRIBUTIONS

Zhao TT: collected the clinical data and wrote the first draft of the manuscript. Liu D: edited the manuscript. Xing Q: was responsible for the overall treatment of the patient and revised the manuscript. Yang XJ: assisted in drafting and revising the manuscript. Yao XF: revised the manuscript critically. Ba Y: revised the manuscript. Zhou HC: revised the manuscript.
